# Analysis of various potential prognostic markers and survival data in clear cell renal cell carcinoma

**DOI:** 10.1590/S1677-5538.IBJU.2015.0521

**Published:** 2017

**Authors:** Tastekin Ebru, Oz Puyan Fulya, Akdere Hakan, Yurut-Caloglu Vuslat, Sut Necdet, Can Nuray, Ozyilmaz Filiz

**Affiliations:** 1 Department of Pathology, Faculty of Medicine, Trakya University, Edirne, Turkey;; 2 Department of Urology, Faculty of Medicine, Trakya University, Edirne, Turkey;; 3 Department of Radiation Oncology, Faculty of Medicine, Trakya University, Edirne, Turkey;; 4 Department of Biostatistics, Faculty of Medicine, Trakya University, Edirne, Turkey

**Keywords:** Carcinoma, Carcinoma, Renal Cell, Survival Rate

## Abstract

**Purpose:**

Clear cell renal cell cancers frequently harbor Von Hippel-Lindau gene mutations, leading to stabilization of the hypoxia-inducible factors (HIFs) and their target genes. In this study, we investigated the relationship between vascular endothelial growth factor (VEGF), HIF-1α, HIF-2α, p53 positivity, microvessel density, and Ki-67 rates with prognostic histopathologic factors (Fuhrman nuclear grade, stage, and sarcomatoid differentiation) and survival in clear cell renal cell carcinomas.

**Material and Methods:**

Seventy-two nephrectomy specimens diagnosed as clear cell renal cell carcinoma between 2000 and 2012 were reevaluated. Immunohistochemically VEGF, HIF-1α, HIF-2α, p53, CD34 (for microvessel density evaluation), and Ki-67 antibodies were applied to the tumor areas. The relationships of these antibodies with prognostic factors and survival rates were evaluated with statistical analyses.

**Results:**

Mean survival time was 105.6 months in patients with ccRCC. Patients with high expression of VEGF, HIF-1α and HIF-2α positivity, a high Ki-67 proliferation index, and a high microvessel density evaluation score had a shorter survival time (p<0.05).

**Conclusions:**

Our findings supported that with the use of these immunohistochemical markers, prognosis of renal cell carcinoma may be predicted at the first step of patient management. New treatment modalities targeted to HIF-1α and HIF-2α might be planned as well as VEGF-targeted therapies in the management of clear cell renal cell carcinomas.

## INTRODUCTION

Renal cell carcinoma (RCC) is the third most common urological malignancy and represents 5% of all cancer diagnoses. Clear cell renal cell cancers (ccRCCs) represent 70% of all renal cancers, and several clinical and histopathologic factors are implicated in the prognosis of renal cancers. Since the World Health Organization updated its classification of kidney tumors in 2004, many studies on histological subtypes, stage, Fuhrman nuclear grade (FNG), prognostic histopathologic factors, and the relationships of these prognostic factors and various immunohistochemical antibodies were conducted. Various studies were conducted to detect the angiogenic and diagnostic factors of ccRCCs and to find new evaluation criteria (1).

Sporadic ccRCC is caused by Von Hippel-Lindau (VHL) tumor suppressor gene mutations located on chromosome 3p in up to 90% of cases. This gene plays a critical role in hypoxia response, including stimulation of neoangiogenesis. According to the most recent studies, common angiogenesis and abnormal blood vessel growth have a direct correlation with the prognosis of renal cell carcinoma (2-5).

The best-documented function of the VHL gene is its role in the oxygen-sensing pathway comprising the substrate recognition component of the E3 ubiquitin ligase complex. This complex targets hypoxia inducible factors (HIFs) for polyubiquitination and proteasomal degradation. The HIF heterodimer can translocate to the nucleus and transactivate the target genes, many of which promote adaptation to acute or chronic hypoxia, including vascular endothelial growth factor (VEGF), which promotes angiogenesis (2, 6). The mutation or inactivation of VHL genes leads to uncontrolled expression of HIF-1α that leads to increased HIF-1α levels in a cell. This complex leads to the transcription of genes that are susceptible to hypoxia and are related to cell survival, regulation of pH levels, glucose metabolism, and angiogenesis, such as VEGF, platelet-derived growth factor (PDGF), transforming growth factor alpha (TGF-α), erythropoietin, and carbonic anhydrase 9 (6). VEGF is the most potent endothelial cell-specific angiogenesis factor. It increases vascular permeability that leads to endothelial cell proliferation, migration, and tube formation (7). Many studies on the influence of VEGF and HIFs on prognosis have been conducted. The relation of these antibodies to targeted therapies, nuclear grading, and tumor size and sarcomatoid differentiation (SD) are increasingly intriguing subjects for studies. These factors offer hints about the progress, strategy, and results of the treatment or chances of relapse. In addition, RCC, a clinically angiogenic activity, has a direct relation with the expression of VEGF. This led to VEGF inhibition-based treatment methods used today against RCC (8).

Immunohistochemically, p53 positivity, and a high Ki-67 proliferating index are associated with cell proliferation. Many studies on the Ki-67 proliferating index and mutant p53 positivity as independent prognostic factors in RCC have been conducted (9). In addition, as an important indicator in RCC prognosis, angiogenesis assessment can be carried out using CD34 antibodies to measure microvessel density (MVD) levels. Recent studies have focused on the importance of these factors in determining the average life expectancy (7, 9, 10).

In this study, we investigated the relationship of VEGF, HIF-1α, HIF-2α, p53 positivity, MVD, and Ki-67 rates with prognostic histopathologic factors (FNG, stage, and SD), and survival in ccRCCs.

## MATERIALS AND METHODS

### Study population and clinical and pathological analysis

The surgical pathology reports of all patients who underwent nephrectomy for RCC between 2000 and 2012 at Department of Pathology, Trakya University Medical Faculty, were reviewed. The follow-up time was a minimum of 2 months and a maximum of 168 months in this study. The surgical pathology reports of all patients who underwent nephrectomy for RCC between 2000 and 2012 at Trakya University Medical Faculty, Department of Pathology were reviewed. The follow-up time was a minimum of 2 months and a maximum of 168 months in this study.

Thirty-two (44.4%) of the patients died during the study and the death reasons for the all patients were clear cell RCC. The pathology reports, as well as the clinical and follow-up data, were retrospectively analyzed. The tumor slides of all patients were reexamined by the Department of Pathology. Histological factors were reevaluated blindly and independently by two pathologists (E.T. and F.O.P.).

### Ethical approval

All procedures performed in studies involving human participants were in accordance with the ethical standards of the institutional and/or national research committee and with the 1964 Declaration of Helsinki and its later amendments or comparable ethical standards (152/2014-17/10). We obtained clinical and pathological data for all enrolled patients from our database and analyzed the results. Informed consent was obtained from all individual participants included in the study.

### Histological evaluation

Tumor grade was based on the Fuhrman nuclear grade system (11), and the tumors were staged according to AJCC/UICC TNM, 7^th^ edition (12). SD was assessed on histologic sections and was graded into two categories, present or absent.

### Immunohistochemical analysis

Before the study, control tissues (from data sheets) were obtained from the archive for each antibody, and then the control staining of these materials was performed. Of these antibodies, Ki-67, p53, and HIF-1α showed nuclear staining, VEGF and CD34 showed cytoplasmic staining, and HIF-2α showed nuclear and cytoplasmic staining. Cytoplasmic staining has been approved as significant for HIF-1α by some researchers (13). Considering the molecular characteristics of HIF-1α and HIF-2α, we used approved positive nuclear staining in this study.

### VEGF evaluation

The immunostaining of VEGF (Clone SP28) was evaluated as a percentage of the cytoplasmic staining pattern in tumor cells. At least 10 high-power fields, including tumors, were evaluated. Moderate cytoplasmic staining was observed in healthy renal tubules (6), and the intensity and percentage of the tumor cells stained with VEGF were evaluated (14). The percentages of VEGF-positive tumor cells were scored as 0 (no staining), 1 (1–25% positive cells), 2 (26–50% positive cells) and 3 (>50% positive cells). The VEGF staining intensity was also scored as 0 (negative), 1 (weak), 2 (intermediate), and 3 (strong). The sum of the percentage and intensity scores was evaluated, and a final score was noted as 0 (negative), 1–2 (weak), 3 (moderate), or 4–6 (strong expression). Cases were divided into two groups, group 1 (score 0–3) and group 2 (score 4–6).

### HIF-1α and HIF-2α evaluations

HIF-1α (Clone H1alpha67) and HIF-2α (Clone D9E3) immunoreactivity was assessed separately for staining distribution and intensity. For the HIF-1α and HIF-2α assessment, the staining intensity was scored as 0 (negative), 1 (weak), 2 (medium), and 3 (strong). The extent of the staining was scored as 0 (0%), 1 (1–25%), 2 (26–50%), 3 (51–75%), and 4 (76–100%) according to the percentages of the positive staining areas in relation to the entire carcinoma area. The sum of the intensity and extent score was used as the final staining score (0–7) for HIF-1α and HIF-2α. Tumors that had a final staining score of 2 and higher were considered positive, and tumors that had a final staining score lower than 2 were considered negative (6, 13).

### p53 evaluation

For p53 (Clone DQ-7) assessment, 1000 tumor cells were evaluated, and cases with nuclear staining were considered positive (9).

### Ki-67 evaluation

For detection of the Ki-67 (Clone SP26) proliferating index, 1000 tumor cells were counted, and the average value was determined statistically (9).

### MVD (CD34 antibody) evaluation

For MVD assessment, the areas of the tumor containing the most capillaries and small venules (i.e., areas of the most intense neovascularization) were examined with a light microscope. Tumors are frequently heterogeneous in microvessel density, but the areas with the highest neovascularization were found by scanning the tumor sections at low power (40X and 100X). CD34 antibody (Clone QBEnd-10) was applied to identify the highest number of discrete microvessels in the tumor areas. Microvessels in sclerotic areas within the tumor, where microvessels are sparse, and immediately adjacent areas of unaffected kidney tissue were not considered in the vessel counts. Any brown-staining endothelial cell or endothelial cell cluster that was clearly separate from adjacent microvessels, tumor cells, and other connective tissue elements was considered a single, countable microvessel. Vessel lumens, although usually present, were not necessary for a structure to be defined as a microvessel, and red cells were not used to define a vessel lumen. Vessel count was performed on a 200X field in five areas, and the average value was determined statistically (10).

### Survival data

Survival information was obtained from the university’s patient follow-up unit.

### Statistical analysis

Patients were classified by their survival status (alive vs. dead). Results are shown as mean±standard deviation, median (minimum–maximum) or number (percentage). The Student t test was used for comparison of age between the alive and dead groups. Gender, FNG, stage, SD, VEGF, HIF-1α, HIF-2α, and P53 values between the alive and dead groups were compared with the chi-square test. The Ki-67 and MVD values between the alive and dead groups were compared with the Mann-Whitney U test. Relationships between VEGF, HIF-1α, HIF-2α, P53, Ki-67, and MVD with FNG, stage, and SD were analyzed with point-biserial correlation analysis. Survival function of patients with ccRCC was analyzed by using the Kaplan-Meier survival analysis according to the stage, FNG, SD, VEGF, and HIF-1α. Then, the log-rank test was used for comparison of survival status. Cut-off values were determined by using the ROC analysis for Ki-67 and MVD, and then the sensitivity, specificity, and area under the curve (AUC) values were calculated based on these cut-off points. A multivariable Cox regression analysis was used to investigate the effect of stage, FNG, SD, VEGF, and HIF-1 alpha on survival.

Statistical analyses were performed using SPSS 20.0 (IBM SPSS Inc., Chicago, IL, USA) and MedCalc11.1.1.0 (MedCalc Software bvba, Ostend, Belgium) statistical software.

## RESULTS

### Demographic data

Seventy-two patients with ccRCC were included in the study. Forty-nine patients were male, and 23 were female (68.1% and 31.9%). Their ages were between 26 and 80 years. Forty (55.6%) of the patients were alive during the study. Patients who were alive had a mean age of 58.48±8.11 years, and deceased patients had a mean age of 65.94±8.65 years. The number of patients according to stage were 25, 17, 21, and 9 in stages 1, 2, 3, and 4, respectively. Most of the patients were localized in FNG 2 (30 patients) and FNG 3 (27 patients). The FNG 1 group included only 5 patients, and the FNG 4 group included 9 patients. Although 11 patient’s tumors showed SD, 61 patient’s tumors did not.

Survival evaluation revealed a general survival rate of 78.21% and a mean survival time of 86.6 months (86.59±4.8) in ccRCC. The median follow-up time was 76 months (min 1.5–max 168.3 months). The mean survival time was 105.6 months in patients with ccRCC. The 1-, 5- and 10-year survival rates were 95.8%, 72.8%, and 48.8% respectively. Survival time was shorter in patients with advanced stage (p<0.001), high grade (p<0.001), and SD positivity (p<0.001). Forty-four of the 72 patients had metastatic ccRCC. According to the medical oncology clinical data, 32 patients were treated with tyrosine kinase inhibitors, and 18 patients received mTOR inhibitors.

**Figure 1:**
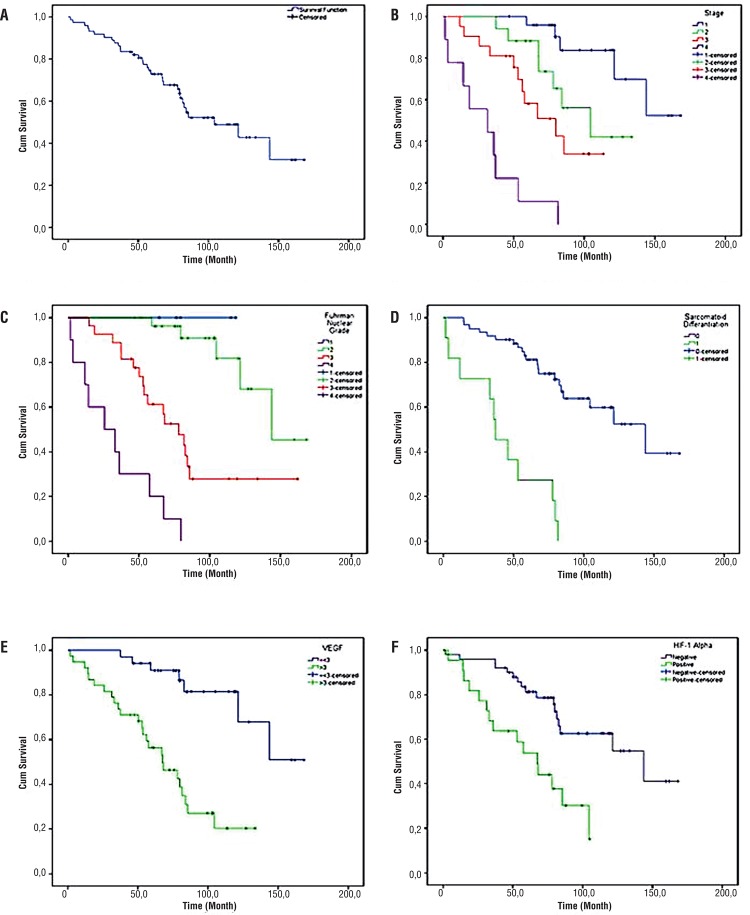
Relationship of histopathological and immunohistochemical characteristics with survive. A) Survival function of patients with RCC, B) Survival function of patients with RCC by stage, C) Survival function of patients with RCC by Fuhrman Nuclear Grade, D) Survival function of patients with RCC by Sarcomatoid differentiation. E) Survival function of patients with RCC by VEGF, F) Survival function of patients with RCC by HIF-1 Alpha.

**Figure 2:**
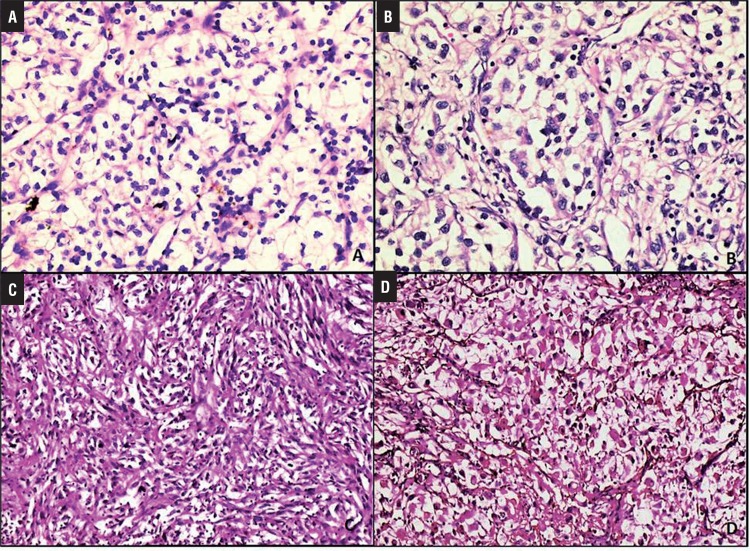
Microscopic features of clear cell renal cell carcinomas. A) Fuhrman grade 1 tumor (H&EX100), B) Fuhrman grade 3 tumor (H&EX200), C and D) Sarcomatoid differentiation in clear cell renal cell carcinoma (H&EX50).

**Figure 3:**
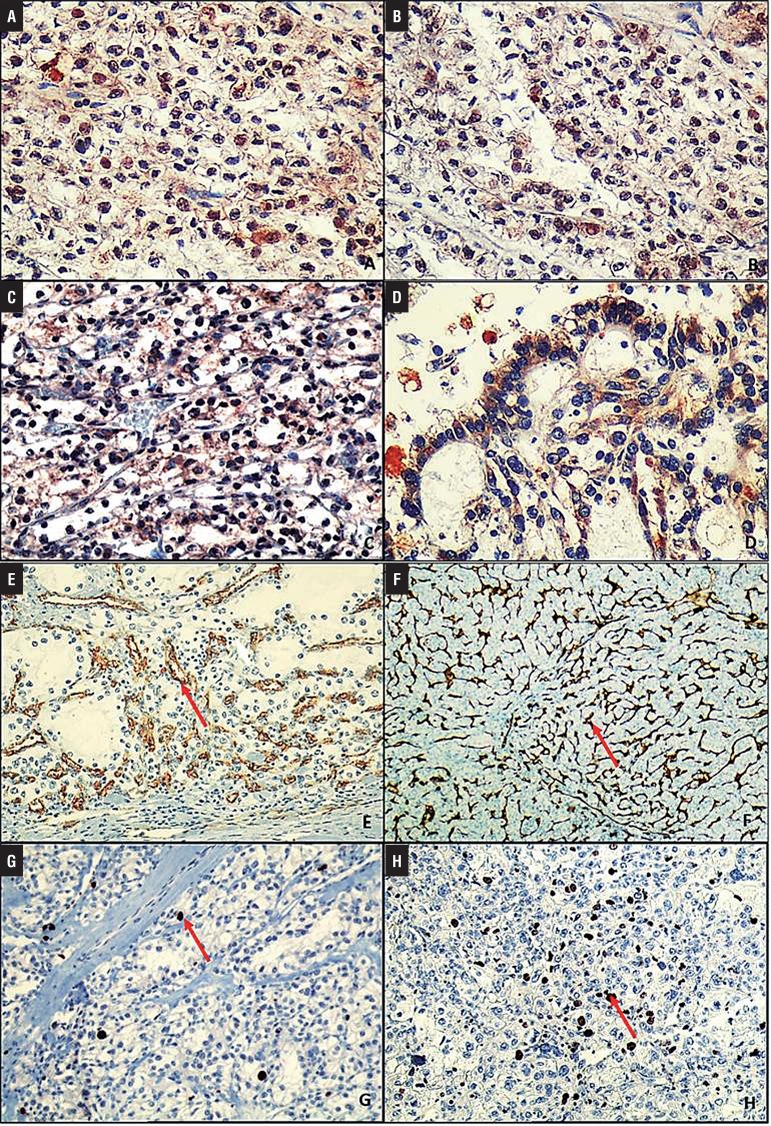
Immunohistochemical staining examples of ccRCCs. A) Widespread, strongly HIF 1α positivity (X400), B) Widespread, moderate HIF 2α positivity (X400), C) Widespread, moderate (X200) and D) focally, strong VEGF positivity (X400), E) Numerous CD34 positive moderate sized and F) small sized vessels (arrow) (X100), G) Nuclear Ki-67 positivity (X200), H) Nuclear p53 positivity (X200) (arrow in G and H: nuclear staining).

Table-1 and Figure-1 show the patient’s demographic data, histopathologic prognostic features (FNG, stage, SD), and their relationships with survival. Immunohistochemical staining features are shown in Figure-2 and listed in Tables 2 and 3; microscopic features and immunohistochemical staining examples of ccRCCs are shown in Figures 2 and 3.

**Table 1:** Clinical and pathological characteristics of patients.

	Alive	Dead	P
**Age (year±SD)**	58.48±8.1	65.94±8.6	<0.001*
**Gender**	**(n/% )**	**(n/% )**	
Male	27 (%68)	22 (%69)	0.910
Female	13 (% 32)	10 (%31)	
**Fuhrman Grade**	**(n/% )**	**(n/% )**	
1	5 (%13)	0 (%0)	<0.001*
2	25 (%62)	5 (%16)	
3	10 (%25)	17 (%53)	
4	0 (%0)	10 (%31)	
**Stage**	**(n/% )**	**(n/% )**	
1	20 (%50)	5 (%16)	<0.001*
2	10 (%25)	7 (%22)	
3	10 (%25)	11 (%34)	
4	0 (%0)	9 (%28)	
**SD**	**(n/% )**	**(n/%)**	
+	0 (% 0)	11 (%34)	<0.001*
-	40 (%100)	21 (%66)	

**SD** = Sarcomatoid differentiation

**Table 2:** Distribution of immunohistochemical characteristics of patients.

	Alive	Dead	p
**VEGF**	**(n/% )**	**(n/% )**	
0-3	27 (%68)	7 (%22)	0.002*
4-6	13 (%32)	25 (%78)	
**HIF-1 alpha**	**(n/% )**	**(n/% )**	
+	7 (%18)	15 (%47)	0.007*
-	33 (%82)	17 (%53)	
**HIF-2 alpha**	**(n/% )**	**(n/% )**	
+	5 (%13)	7 (%22)	0.289
-	35 (%87)	25 (%78)	
**P53**	**(n/% )**	**(n/% )**	
+	1 (%3)	5 (%13)	0.041*
-	39 (%87)	27 (%87)	
**Ki-67** (median/min-max)	70.5(10-289)	145.5 (27-453)	<0.001*
**MVD** (median/min-max)	141.5 (91-400)	280 (98-250)	<0.001*

**VEGF** = Vascular endothelial growth factor; **HIF** = Hypoxia inducible factor; **MVD** = Micro vessel density

**Table 3:** Distrubution of immunohistochemical markers between the stages, Fuhrman nuclear grade and sarcomatoid differentiation.

		HIF-1 alpha		HIF-2 alpha		VEGF						

		(-)	(+)	(-)	(+)	0	1	2	3	4	5	6
**STAGE (n)**	**1** (n=25)	4	21	4	21	1	11	23	0	0	0	0
	**2** (n=17)	5	12	4	13	0	0	0	9	8	0	0
	**3** (n=21)	8	13	3	18	0	0	0	0	10	9	2
	**4** (n=9)	5	4	1	8	0	0	0	0	0	3	6
**FNG (n)**	**1** (n=5)	2	3	2	3	1	1	3	0	0	0	0
	**2** (n=30)	5	25	2	28	0	9	9	4	5	3	0
	**3** (n=27)	8	19	6	21	0	1	1	5	11	6	3
	**4** (n=10)	7	3	2	8	0	0	0	0	2	3	5
**SD (n)**	**(-)** (n=61)	17	44	11	50	1	11	13	8	15	9	4
	**(+)** (n=11)	5	6	1	10	0	0	0	1	3	3	4

**FNG** = Fuhrman nuclear grade; **SD** = Sarcomatoid differentiation; **VEGF** = Vascular endothelial growth factor; **HIF** = Hypoxia inducible factor; **SD** = Sarcomatoid differentiation

### VEGF and survival status

With the VEGF antibody, only 4 cases did not show staining, while the others had different levels of staining. Twenty-seven (37.5%) cases had strong staining for VEGF, and only 6 of them (22.22%) were survivors. We observed a direct relation between the VEGF score and the stage, Fuhrman nuclear grade, and SD positivity (r=0.935, p<0.001; r=0.692, p<0.001; r=0.394, p<0.001, respectively). We observed that although the VEGF stain score increased, the mean survival rates decreased. The mean survival time for cases with a VEGF stain score of 1 was 142.91 months, on average, in cases with score 2, 106.3 months, and in cases with score 3, as low as 56.09 months (p<0.01; see Table-4).

**Table 4:** Comparison of the pathological and immunohistochemical characteristics.

	Prognostic factor		

	Pathological characteristic	r	p
**VEGF**	Fuhrman	0.692	<0.001*
	Stage	0.935	<0.001*
	SD	0.394	<0.001*
**HIF-1 alpha**	Fuhrman	0.264	<0.05*
	Stage	0.277	<0.05*
	SD	0.137	0.25
**HIF-2 alpha**	Fuhrman	0.073	0.54
	Stage	0.043	0.72
	SD	0.086	0.47
**p53**	Fuhrman	0.263	<0.05*
	Stage	0.086	0.47
	SD	0.290	<0.05*
**Ki-67**	Fuhrman	0.644	<0.001*
	Stage	0.738	<0.001*
	SD	0.349	<0.01*
**MVD**	Fuhrman	0.652	<0.001*
	Stage	0.640	<0.001*
	SD	0.347	<0.01*

**VEGF** = Vascular endothelial growth factor; **HIF** = Hypoxia inducible factor; **MVD** = Micro vessel density; **SD** = Sarcomatoid differantiation

### HIF-1α and survival status

Twenty-two cases (30.5%) showed a positive reaction with HIF-1α; 7 of these cases (31.8%) were survivors. We observed a direct correlation between HIF-1α positivity and FNG (r=0.264; p<0.05) and stage (r=0.277; p<0.05). No statically significant relation between HIF-1α and SD was observed (p=0.25). A shorter survival time was present in cases of HIF-1α positivity. Cases with HIF-1α positivity had an average survival time of 63 months, while cases with negativity had an average survival time of 120 months (p<0.05; see Table-4).

### HIF-2α and survival status

Twelve cases (16.6%) showed a positive reaction with HIF-2α, and 7 of these cases (31.8%) were survivors. No statically significant relation between HIF-2α, FNG, stage, and SD was found. We observed a shorter average survival time for patients with HIF-2α positivity. The mean survival time for the HIF-2α-positive cases was 88 months and for the negative cases was 107 months (p<0.05; see Table-4).

### p53 and survival status

Six cases (8.3%) showed a positive reaction for p53, and only 1 of these cases (16.7%) was a survivor. A relation and a direct correlation were observed between p53 positivity and FNG (r=0.263; p<0.05). A similar relation and correlation were also observed between p53 positivity and SD (r=0.290, p<0.05). We observed a shorter average survival time for patients with p53 positivity. The p53 positive cases had a mean survival time of 42 months, while negative cases had a mean survival time of 111 months (p<0.05; see Table-4).

### Ki-67 and survival status

The Ki-67 proliferation index and FNG, stage, and SD had a positive statically significant relationship (r=0.644, p<0.001; r=0.738 p<0.001; r=0.349, p<0.01, respectively). The mean Ki-67 value for was 57.5 for deceased patients and 27.6 for survivors (p<0.001; see Table-4).

### MVD and survival status

MVD and FNG, stage, and SD had a positive statically significant relationship (r=0.652, p<0.001; r=0.640, p<0.001; r=0.347, p<0.01, respectively). The mean MVD value was 46.5 for deceased patients and 28.5 for survivors (p<0.001; see Table-4). The effect of stage, FNG, and SD on survival status was evaluated with multivariable Cox regression analysis. The survival rate was negatively affected by stage 1-4, FNG 3 and 4, and SD positivity (Table-5).

**Table 5:** The effect of stage, FNG and SD on survival.

Cox regression-Multivariate						

		HR	95% CI for HR		p	
**Stage**	I-II	reference				
	III-IV	2.694		1.163 – 6.241		0.021
**FNG**	I-II	reference				
	III-IV	5.286		1.885 – 14.819		0.002
**SD**	(-)	reference				
	(+)	2.575		1.111-5.971		0.027

**FNG** = Fuhrman nuclear grade; **SD** = Sarcomatiod differentiation

### Immunohistochemical data inter-correlation

VEGF had statically significant relationships with HIF-1α (r=0.566; p<0.001), MVD (r=0.669; p<0.01), and the Ki-67 proliferation index (r=0.764; p<0.001). In addition, HIF-1α had a similar statically significant relationship with HIF-2α (r=0.270; p<0.05), p53 (r=0.234; p<0.05), the Ki-67 proliferation index (r=0.350, p<0.05), and MVD (r=0.399, p<0.05). The Ki-67 proliferation index and MVD also had a statically significant relationship (r=0.618; p<0.01; see Table-4).

**Figure 4:**
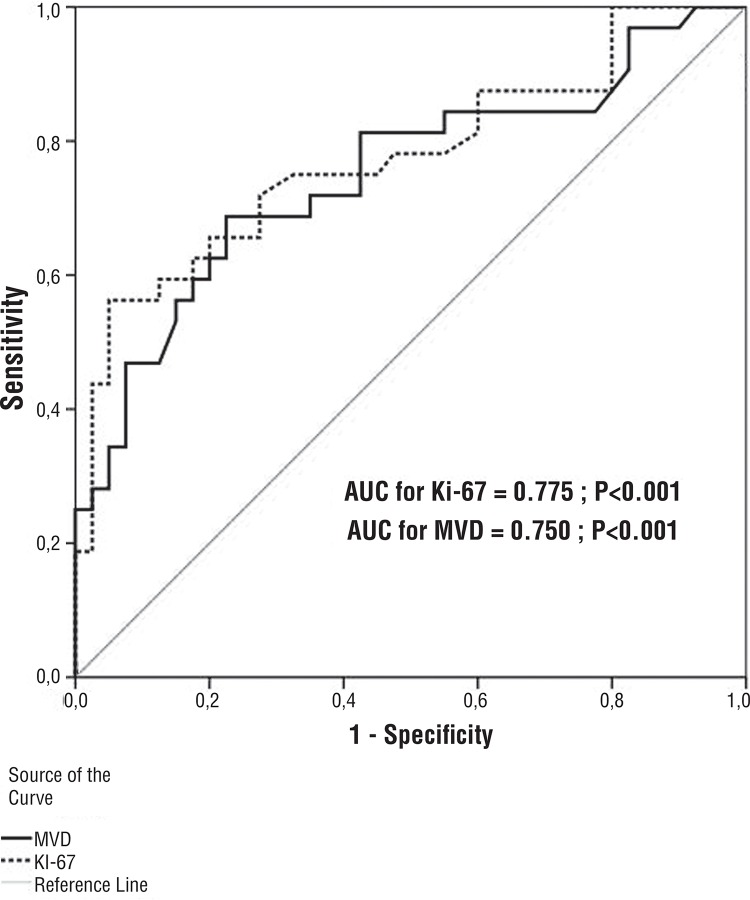
ROC Curve analysis for Ki-67, and MVD

We evaluated the mortality difference for Ki-67 and MVD with ROC analysis. At the time of diagnosis, Ki-67 had a cut-off point >132 at 56.2% sensitivity and 95% specificity. MVD had a cut-off value of >180 and, at this point, 68.7% sensitivity and 77.5% specificity (Figure-4).

## DISCUSSION

Genetic and molecular prognostic factors specific to disease in RCC are an important topic for all current research related to this field. Recent studies have increased our understanding of the molecular biology of RCC, leading to the development of better-guided molecular treatment methods. Based on this information, immunohistochemical indicators are becoming more common of a subject for studies to predict the prognosis of patients with ccRCC and to determine treatment methods (9).

In this study, immunohistochemically VEGF, HIF-1α, HIF-2α, p53, CD34 (for MVD evaluation), and Ki-67 antibodies were applied to the tumor areas, and the relationships of these antibodies with prognostic factors (FNG, stage, and SD) and survival rates were evaluated. We observed that VEGF and HIF-1α proteins that play an important role in tumor angiogenesis, p53 gene mutation related to apoptosis, the Ki-67 proliferation index, prognostic properties of the tumor (FNG, stage, SD) related to each other or independently affect patient’s survival may lead to poor prognosis. The development of new treatment methods and agents will lead to new horizons, especially in metastatic RCCs.

### VEGF and prognosis

Angiogenesis-related growth factors, in which the Von Hippel-Lindau gene plays a role regarding regulation, are the most important objectives in the treatment of ccRCCs. VEGF is a critical cytokine regarding tumor angiogenesis. RCC, a clinically vascular tumor, has a direct correlation with VEGF expression. For this reason, ccRCC and VEGF inhibition are associated in treatment. Chang et al. reported higher levels of VEGF expression in ccRCC than in healthy kidney parenchyma (15). Furthermore, recent studies have shown correlations between VEGF expression and microvascular density, tumor size, nuclear grade, stage, and prognosis in ccRCC (6, 16).

Recent studies on ccRCC showed that expression of VEGF was directly related to stage, capsular invasion, size, and nuclear grade in ccRCC (8, 16, 17). In the present study, consistent with other studies, patients with a high FNG and stage and with SD, had high staining scores.

Yilmazer et al. (18) showed that the expression of VEGF was correlated with vascular density. Cases with high VEGF stain scores had also high MVD. Our findings are compatible with the finding as we observed a positive correlation between MVD and the VEGF staining score. Burgesser et al. (6) reported that the expression of VEGF was directly related to the proliferation index. Our results were compatible with theirs. Samples with a high stain score had low average survival rates. These findings show that VEGF related to a poor prognosis in many tumors is also related in the same way with ccRCC.

## HIF-1α, HIF-2α and prognosis

Because HIF-α activates related genes in the regulation of angiogenesis, glucose metabolism, pH control, epithelial proliferation, and apoptosis, it is related to progression in many types of cancer. In the kidney, a substantial amount of HIF-1α is released from many cells; HIF-2α is released from interstitial fibroblast and endothelial cells (19). Many studies, including Byun et al. (20), have revealed significant associations between HIF-2α and tumor stage. Dornbusch et al. (2) reported similar results in 2013. However, Haase (21) reported that in the kidney’s normal tubular epithelial cells gene expression related to HIF is more under HIF-1α control. According to their study on RCC tumors, with VHL mutations, while the HIF-2α level is high, HIF-1α is not. Sowter et al. (19) conducted a study on the role of HIF proteins and demonstrated that in RCC the HIF-2α network has a fundamental role in many stages of cancer development. Raval et al. (22), in a study of the effects of HIF subgroups in VHL-related RCC, emphasized that HIF-2α has a tumor-growing effect and HIF1-α stops tumor growth. In the present study, similar to the Byunn et al. (20) results, we found that although RCCs with high HIF-1α staining scores are related to FNG, stage, and SD, the HIF-2α staining scores are not. In the HIF-1α- and HIF-2α-positive samples, the average survival rate decreased. In the literature review, Lidgren et al. (13) tracked HIF-1α positivity in RCC samples with low degree, early stage, and high survival rates and accepted HIF-1α positivity as an indicator of a good prognosis; while Klatte et al. (23) found HIF-1α positivity in RCC with renal cells and found no relationship between stage, FNG, and HIF-1α staining. However, HIF-1α and HIF-2α positivity was found to be related to a poor prognosis and low survival rates in many tumor types and ccRCCs in the literature, as in the present study.

According to various studies about angiogenesis in the available literature, VEGF and HIF-1α are the most important. However, the results are controversial and inconsistent (19, 20).

Most studies also focused on tumor microvascular density, because it is directly associated with the expression of angiogenic factors. Minardi et al. reported a direct relationship between HIF-1α expression and vascular density (24). The data from the present study were compatible with this report. HIF-1α expression was correlated with higher MVD and Ki-67 proliferation index values. One point that should be discussed, which is an issue of conflict in different publications, is the staining property of HIF-1α. Taking molecular properties into account, HIF-1α is activated only in the nucleus and is subject to translocation (3). In some studies, cytoplasmic staining and nuclear staining are considered positive, while in other studies only cytoplasmic staining is positive (13). Some studies have shown that staining patterns differ between tumors and non-tumor tissues or in VHL mutant or non-mutant samples. The differences in the literature indicate that the staining pattern continues to be a matter of debate (25).

### p53 and prognosis

p53 expression in RCCs is disputed. In the literature (9), p53 expression is related to a poor prognosis and SD. In the findings of the present study, high-grade p53 positivity was highly correlated with SD, one of the indicators of a poor prognosis in ccRCC. No relation was observed between p53 positivity and stage. The antibody’s positivity is not related to MVD and the proliferation index. Phouc et al. (26) demonstrated that a high staining score of the p53 gene in RCCs is inversely related to survival. Olumi et al. (27), in a study with a low number of samples, did not find a relationship between the p53 gene in RCCs and survival; however, the data for p53 staining in the present study show a statically significant relationship between p53 positivity and survival. The p53-positive samples had lower survival rates and lower average lifetime.

### Ki-67 and prognosis

Studies on the effects of Ki-67, which is an indicator of active cell proliferation, on prognosis in patients with RCC have been conducted. Bakır and Özekinci (28) reported a relationship between high Ki-67 positivity and advanced pathological stage and poor prognosis. They reported that more than 9% proliferation with Ki-67 is an independent indicator of poor prognosis. Itoi et al. (29) reported that Ki-67 is an independent prognostic factor in RCC while p53 is not a sufficient prognostic indication. Our findings showed that the Ki-67 proliferation index and the stained nucleus number are directly proportionate and are related to stage, FNG, and SD. An increase in the Ki-67 proliferation index is also accompanied by a decrease in survival time. This finding was statistically significant and in agreement with the literature.

### MVD (CD34 antibody) and prognosis

CD34 is a molecule related to the abluminal endothelial microprocess that causes vascular sprouting in the tumor’s stroma in the angiogenesis stage, used in measuring microvessel density. The CD34 antibody is better than other antibodies for use in microvessel measurements to detect prognosis (8). Yılmazer et al. (18) reported that in advanced stage tumors, the vascular density was high (p<0.05). Kavantzas et al. (7) showed that higher vascular density was related to a higher nuclear grade. However, MacLennan (4) found no significant correlation between vascular density and tumor stage. Nativ et al. (30) showed that vascular density was lower in tumors with a higher FNG. The present data show that in patients with indicators of poor prognosis such as a high FNG, advanced stage, and SD MVD was high. Bürgesser et al. reported that tumors with higher HIF-1α expression had higher MVD (6). The present data are comparable with these findings. Bürgesser et al. reported that higher vascular density was related to lower survival rates (6). The data of the present study confirm theirs.

### MVD and Ki-67 values and cut-off values

The cut-off value we observed for MVD and the Ki-67 evaluation is a new and low-sensitive suggestion. However, new studies with a higher number of patients the importance of these findings must be noted. The cut-off value detected in larger studies might be helpful in treatments for breast carcinomas, for example.

RCC is very resistant to standard chemotherapy. Biological and immune-based therapies and treatment options for patients with RCC are limited, and the response rates are low. Immunotherapy with interferon-α and interleukin-2 once represented the standard treatment for RCC; however, both are associated with substantial toxicity, and response rates are limited (2). Understanding of the pathogenesis of ccRCC has facilitated the development of new RCC-targeting therapies. The discovery of VHL inactivation and HIF activation of genes and other pathways that are important for tumor progression has aided the development of new drugs that target angiogenesis and proliferation pathways. Drugs that target the VEGF pathway have been approved for RCC. Although these new agents may improve survival rates, none are curative. At present, no predictive biomarkers have been established for these drugs. Given that these drugs inhibit this pathway at the protein level, target protein expression might be associated with response to therapy. Some attempts have been made to develop predictive biomarkers that are primarily centered on VHL mutations and the HIF and VEGF levels.

Another aspect of this subject is that showing protein expression levels with immunohistochemical staining might be a supportive method for predicting RCC survival features, treatment regimens, and management of patient. An evaluation of the literature on RCCs showed the relationships of angiogenetic and prognostic factors, with each other and with immunohistochemical indicators, are debatable.

In this study, we suggested that VEGF staining scores, HIF-1α, HIF-2α, p53 positivity, MVD, and Ki-67 counts may be used for the prediction of prognosis in patients with ccRCC. In addition, new treatment regimens targeted to this pathway may be planned. Especially, these therapeutic agents may be a gleam of hope for patients with metastatic ccRCC.

## CONCLUSIONS

In conclusion, although there have been different reports, the correlation between HIF-1α, HIF-2α, VEGF, and p53 positivity, high MVD, and the Ki-67 proliferation index with prognostic histopathological features and survival rates was consistent with most of the findings in the literature. Differences in the staining scores of immunohistochemical antibodies provide preliminary information about patient survival. In light of these findings, new target-oriented treatments may be improved to target the HIF-1α and HIF-2α pathway, as well as VEGF-targeted therapies that were used. In the future, immunohistochemical evaluation criteria should be standardized, new studies should be planned with comprehensive case series, and results should be supported with molecular studies. Prospective and more standardized studies should be planned to specify the role of angiogenic factors in ccRCC and the results standardized for the prediction of the treatment and management of disease.
